# High-throughput sequencing of CD4^+^ T cell repertoire reveals disease-specific signatures in IgG4-related disease

**DOI:** 10.1186/s13075-019-2069-6

**Published:** 2019-12-19

**Authors:** Liwen Wang, Panpan Zhang, Jieqiong Li, Hui Lu, Linyi Peng, Jing Ling, Xuan Zhang, Xiaofeng Zeng, Yan Zhao, Wen Zhang

**Affiliations:** 1 Department of Rheumatology, Peking Union Medical College Hospital, Chinese Academy of Medical Science & Peking Union Medical College, Key Laboratory of Rheumatology and Clinical Immunology, Ministry of Education, No.41 Da Mu Cang, Western District, Beijing, 100032 People’s Republic of China; 2 Department of General Surgery, Ruijin Hospital, School of Medicine, Shanghai Jiao Tong University, Shanghai, China; 3 Tsinghua University School of Medicine, Beijing, China

**Keywords:** IgG4-related disease, CD4^+^ T cells, TCR repertoire, Complementarity determining region 3, Antigen

## Abstract

**Background:**

CD4^+^ T cells play critical roles in the pathogenesis of IgG4-related disease (IgG4-RD). The aim of this study was to investigate the TCR repertoire of peripheral blood CD4^+^ T cells in IgG4-RD.

**Methods:**

The peripheral blood was collected from six healthy controls and eight IgG4-RD patients. TCR β-chain libraries of CD4^+^ T cells were constructed by 5′-rapid amplification of cDNA ends (5′-RACE) and sequenced by Illumina Miseq platform. The relative similarity of TCR repertoires between samples was evaluated according to the total frequencies of shared clonotypes (metric F), correlation of frequencies of shared clonotypes (metric R), and total number of shared clonotypes (metric D).

**Results:**

The clonal expansion and diversity of CD4^+^ T cell repertoire were comparable between healthy controls and IgG4-RD patients, while the proportion of expanded and coding degenerated clones, as an indicator of antigen-driven clonal expansion, was significantly higher in IgG4-RD patients. There was no significant difference in TRBV and TRBJ gene usage between healthy controls and IgG4-RD patients. The complementarity determining region 3 (CDR3) length distribution was skewed towards longer fragments in IgG4-RD. Visualization of relative similarity of TCR repertoires by multi-dimensional scaling analysis showed that TCR repertoires of IgG4-RD patients were separated from that of healthy controls in F and D metrics. We identified 11 IgG4-RD-specific CDR3 amino acid sequences that were expanded in at least 2 IgG4-RD patients, while not detected in healthy controls. According to TCR clonotype networks constructed by connecting all the CDR3 sequences with a Levenshtein distance of 1, 3 IgG4-RD-specific clusters were identified. We annotated the TCR sequences with known antigen specificity according to McPAS-TCR database and found that the frequencies of TCR sequences associated with each disease or immune function were comparable between healthy controls and IgG4-RD patients.

**Conclusion:**

According to our study of CD4^+^ T cells from eight IgG4-RD patients, TCR repertoires of IgG4-RD patients were different from that of healthy controls in the proportion of expanded and coding degenerated clones and CDR3 length distribution. In addition, IgG4-RD-specific TCR sequences and clusters were identified in our study.

## Background

IgG4-related disease (IgG4-RD) is a newly recognized clinical entity mainly affecting middle-aged to elderly males, characterized by immune-mediated fibro-inflammatory process. Pathologic features of IgG4-RD include dense lymphoplasmacytic infiltration enriched in IgG4-positive plasma cells, storiform fibrosis, and obliterative phlebitis [1, 2]. The pathogenesis of IgG4-RD remains unclear. It has been proposed that chronic antigen stimulation induces activation, clonal expansion, and class switching of IgG4^+^ plasmablasts/plasma cells in a T follicular helper cell (Tfh2)-dependent manner, and the plasmablasts/plasma cells present antigens and activate CD4^+^ cytotoxic T cells (CTLs), which undergo oligoclonal expansion and drive inflammatory and fibrotic processes that characterize IgG4-RD [3–7]. Therefore, CD4^+^ T cells and B cells play central roles in the pathogenesis of IgG4-RD.

The vast majority of T cells express αβ T cell receptors (TCRs), which interacts with peptide-MHC complex presented by antigen-presenting cells [8, 9]. During the development of T cells, TCRs are randomly generated through VJ recombination (α chain) or VDJ recombination (β chain), followed by deletion or insertion of non-template nucleotides at junction sites. Then, T cells are subjected to positive and negative selection in the thymus [8, 10]. The diversity of TCRs is predominantly confined to the complementarity-determining regions (CDR). CDR1 and CDR2 domains are encoded by germline V gene segments, while CDR3 domains, the region that directly contacts with peptide antigen, comprise the VJ junction (α chain) or VDJ junction (β chain). Subsequently, CDR3 domains are highly diverse, allowing the recognition of various antigens [11].

The analysis of the TCR repertoire has been challenging due to its enormous diversity. During the past decades, the TCR repertoire was analyzed by CDR3 spectratyping, which involves amplification of CDR3 by RT-PCR using V and J gene-specific primers, and separating the amplicons by polyacrylamide gel electrophoresis [12, 13]. Nowadays, with the advances in high-throughput sequencing technologies, it is possible to sequence millions of TCR clones simultaneously, so that the full TCR repertoire can be analyzed at single-cell resolution, which enables inspection of the adaptive immune system in details [14].

Here, using the approach of high-throughput sequencing, we studied the TCR repertoire of peripheral blood CD4^+^ T cells from IgG4-RD patients in depth. We compared the characteristics of CD4^+^ T cell repertoire between healthy controls and IgG4-RD patients, including expansion and coding degeneracy levels of each clonotype, CDR3 length distribution, and usage of TRBV and TRBJ genes. In addition, we analyzed relative similarities of TCR repertoires between individuals, identified IgG4-RD-specific CDR3 amino acid sequences, and clustered the TCR clonotypes based on sequence similarities to reveal disease-specific clusters. Finally, we analyzed the antigen specificities of TCR clonotypes according to the McPAS-TCR database.

## Methods

### Patients and healthy controls

We included eight newly diagnosed and untreated IgG4-RD patients, and six healthy controls matched for sex, age, and ethnicity. All of the enrolled patients satisfied the 2011 comprehensive diagnostic criteria for definite IgG4-RD [15]. The patients with infectious diseases, malignancies, other rheumatic diseases, or conditions that could mimic IgG4-RD were excluded. All of the enrolled individuals claimed no history of infection or vaccination within 6 months before recruitment. Whole blood samples were collected at Peking Union Medical College Hospital, between May 2017 and Feb 2018. The study protocol was approved by the Ethics Committee of Peking Union Medical College Hospital. All enrolled participants consented to attend this cohort study and signed written informed consent.

### CD4^+^ T cell isolation and RNA extraction

Peripheral blood mononuclear cells (PBMCs) were isolated from fresh blood by standard Ficoll-Hypaque procedures. CD4^+^ T cells were enriched by positive magnetic-bead selection (Miltenyi, Gladbach, Germany) according to the manufacturer’s instructions. RNA was extracted from CD4^+^ T cells using TRIzol (Invitrogen).

### TCR β-chain library preparation and high-throughput sequencing

TCR β-chain sequences were amplified by 5′-rapid amplification of cDNA ends (5′-RACE), using SMARTer® RACE cDNA Amplification Kit (Clontech). The total RNA input was 1 μg. UPM primer was used as 5′ primer, and TRBC-specific primer, with the sequence “TCTGATGGCTCAAACACAGCGACCT,” was used as 3′ primer. PCR reaction contained 3 μl cDNA, 20 pmol 5′ and 3′ primers, 4 μl 2.5 mM dNTPs, 2.5 U pfu polymerase, and 10 μl 5 × pfu buffer, with the final volume of 50 μl. PCR conditions were 95 °C for 4 min (min), followed by 25 cycles of 94 °C for 30 s, 58 °C for 30 s, and 72 °C for 10 s, and a final extension of 72 °C for 5 min. The amplified TCR β-chain products were then cooled to 4 °C. TCR β-chain sequencing libraries were constructed with NEBNext® Ultra™ DNA Library Prep Kit for Illumina (NEB) and underwent quality control using Bioanalyzer High Sensitivity DNA chip (Agilent). TCR β-chain libraries were sequenced on Illumina Miseq platform (2 × 300 bp).

### Bioinformatics analysis

Raw data were processed by Cutadapt software (v1.9.1) [16] to remove adapter sequences and the bases with quality lower than 20. Paired-end reads were merged into one contig sequence by FLASH software (v1.2.11) [17]. Using MiXCR software (v2.0.2) [18], the clean reads were aligned to human TRBV, TRBD, and TRBJ reference sequences, and TCR clonotypes were assembled, with corresponding CDR sequences. TCR repertoire diversity was assessed by the Shannon-Wiener index [9]. The coding degeneracy level was evaluated for each CDR3 amino acid clonotype, calculated as the number of unique nucleotide sequences encoding each CDR3 amino acid sequence [19]. Dimensionality reduction was performed by Barnes-Hut implementation of t-distributed stochastic neighbor embedding (t-SNE) using Rtsne package [20] (1000 iterations, perplexity parameter of 4, trade-off *θ* of 0.5) and visualized by plotting each event by its t-SNE dimension 1 and dimension 2 in a dot plot.

TCR repertoire similarities between individuals were evaluated by the following metrics using VDJtools [21]: (1) geometric mean of total frequencies of shared clonotypes (metric F), (2) Pearson correlation of frequencies of shared clonotypes (metric R), and (3) normalized number of shared clonotypes (metric D). The repertoire similarities were then visualized by multi-dimensional scaling (MDS) analysis. For TCR network construction, R package “stringdist” [22] was used to calculate Levenshtein distances between each two CDR3 amino acid sequences, and the network figures were made by Cytoscape (http://www.cytoscape.org/) [23]. IgG4-RD-specific clusters were identified in TCR networks. To annotate the TCR clonotypes with known antigen specificity, we referred to the McPAS-TCR database [24]. We annotated all the TCR clonotypes in our dataset of which the Levenshtein distance with the CDR3 sequences in the McPAS-TCR database equals to 0. Detailed information about the calculation of the Shannon-Wiener index, the evaluation of TCR repertoire similarities, the definition of IgG4-RD-specific clusters, and the annotation of TCR clonotypes were provided in Additional file 1. R package “ggplot2” [25], “circlize” [26], and “VennDiagram” [27] were used to plot the figures.

### Statistical analysis

Differences between the groups were analyzed by Welch’s *t* test for the variables following a normal distribution and the Mann-Whitney *U* test for the variables not following a normal distribution. Correlations between nonnormally distributed variables were analyzed by the Spearman rank correlation test. Outliers identified in this study fulfilled both the Dixon criterion and the Grubbs criterion. Bootstrap resampling was conducted with the following procedures: First, we generated 1000 bootstrap samples by randomly sampling the data from a healthy control or IgG4-RD patients with replacement. Each bootstrap sample had the same sample size as the original dataset. Then, we calculated the mean values in bootstrap samples, analyzed the distribution of bootstrap means according to density plot, and estimated the mean and 95% confidence interval accordingly. The existence of outliers was assessed by bootstrap-based outlier detection plot (Bootlier plot), which is constructed by bootstrapping the statistic “mean-trimmed mean” (MTM) and plotting its distribution [28]. The existence of outliers was indicated by the multimodality of Bootlier plot and tested by the Bootlier test, according to the methodology developed by Candelon and Metiu [29]. We performed a nonparametric bootstrap *t* test with a pooled resampling method for group comparison, according to the recommendation by Dwivedi et al. for small sample size studies [30]. For multiple comparison, false discovery rate (FDR) control was performed by the Benjamini-Hochberg procedure. Data analysis was performed by R studio (v3.4.0) and GraphPad Prism 7 software. *p* < 0.05 was considered statistically significant.

## Results

The demographic and clinical characteristics of the individuals enrolled in this study were summarized in Table 1. A detailed description of the total number of raw reads, filtered reads, aligned TCR sequences, and unique clonotypes of each sample was displayed in Additional file 2. 244,175 ± 47,618 TCR sequences were obtained from each individual. Based on distinct CDR1, CDR2, and CDR3 nucleotide sequences, 35,099 ± 9319 clonotypes were identified in each sample. The TCR clonotypes identified in each sample were summarized in Additional file 3.

**Table 1:** Demographic and clinical characteristics of the individuals enrolled in this study

ID	Sex/age	Organ involvement	IgG4-RD RI	Serum IgG4 (mg/L)
HC-1	M/58	–	–	–
HC-2	M/63	–	–	–
HC-3	F/69	–	–	–
HC-4	M/28	–	–	–
HC-5	M/53	–	–	–
HC-6	M/61	–	–	–
PT-1	M/34	Thyroid	4	3450
PT-2	M/61	Salivary glands + retroperitoneum + large artery + lymph nodes + sinus	19	21,000
PT-3	M/56	Lacrimal glands + salivary glands	8	6010
PT-4	M/62	Lacrimal glands + salivary glands + retroperitoneum + lung + large artery + lymph nodes + sinus	25	44,500
PT-5	M/63	Pancreas + prostate + lymph nodes	13	13,600
PT-6	M/48	Pancreas + prostate	12	9300
PT-7	M/61	Lacrimal glands + salivary glands + lung + sinus	16	32,500
PT-8	F/73	Lacrimal glands + ocular adnexa + sinus + retroperitoneum + bladder + lymph nodes	25	23,400

*HC* healthy control, *PT* IgG4-RD patient, *RI* responder index

### Clonal expansion and coding degeneracy of CD4^+^ T cells in healthy controls and IgG4-RD patients

**Fig. 1:**
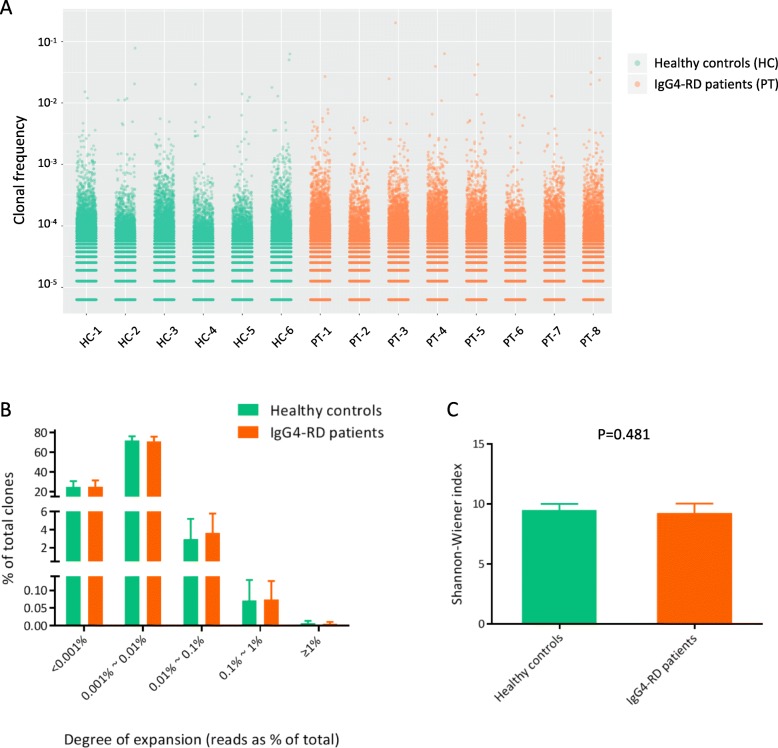
Clonal expansion and diversity of CD4^+^ T cell repertoire of healthy controls and IgG4-RD patients. The TCR repertoires of all the individuals were downsampled to the same number of reads (158,416 TCR sequences per sample). **a** Scatterplot showing the frequencies of all the TCR clones in each sample. Each dot represents one clone. **b** Frequency distribution of CD4^+^ T cell clones in healthy controls and IgG4-RD patients. Data were represented as mean and standard deviation (sd). **c** Shannon-Wiener index as quantification of TCR repertoire diversity of CD4^+^ T cells. Data were represented as mean and sd. Difference between groups was analyzed by Welch’s *t* test

To avoid the potential influence of sequencing depth on the analysis of clonotype frequency and diversity, the TCR repertoires of all the individuals involved were downsampled to the same number of reads (158,416 TCR sequences per sample). The degree of expansion of CD4^+^ T cell clones was assessed by the frequency of clones within a sample. Several clearly expanded CD4^+^ T cell clones were detected in the background of hundreds of low-frequency clones in each sample (Fig. 1a). In Fig. 1b, the frequencies of CD4^+^ T cell clones in both healthy controls and IgG4-RD patients showed a right-skewed distribution, in which the majority of clones were of low frequency (< 0.01%). Therefore, we defined the clones with a frequency of ≥ 0.01% to be expanded clones; and the clones with a frequency of ≥ 0.1% to be highly expanded clones. The proportion of both expanded clones and highly expanded clones were comparable between healthy controls and IgG4-RD patients (proportion of expanded clones: healthy controls—3.05% ± 2.28% [outlier included], 2.18% ± 0.92% [HC-1 excluded as outlier]; IgG4-RD patients—3.72% ± 2.19%; Welch’s *t* test, *p* = 0.589 [outlier included], *p* = 0.108 [outlier excluded]). The proportion of highly expanded clones: healthy controls, 0.080% ± 0.059%; IgG4-RD patients, 0.081% ± 0.054%; Welch’s *t* test, *p* = 0.963). We further evaluated the diversity of CD4^+^ T cell repertoire of each individual based on the Shannon-Wiener index and found that the TCR repertoire diversity was comparable between healthy controls and IgG4-RD patients (Fig. 1c).

**Fig. 2:**
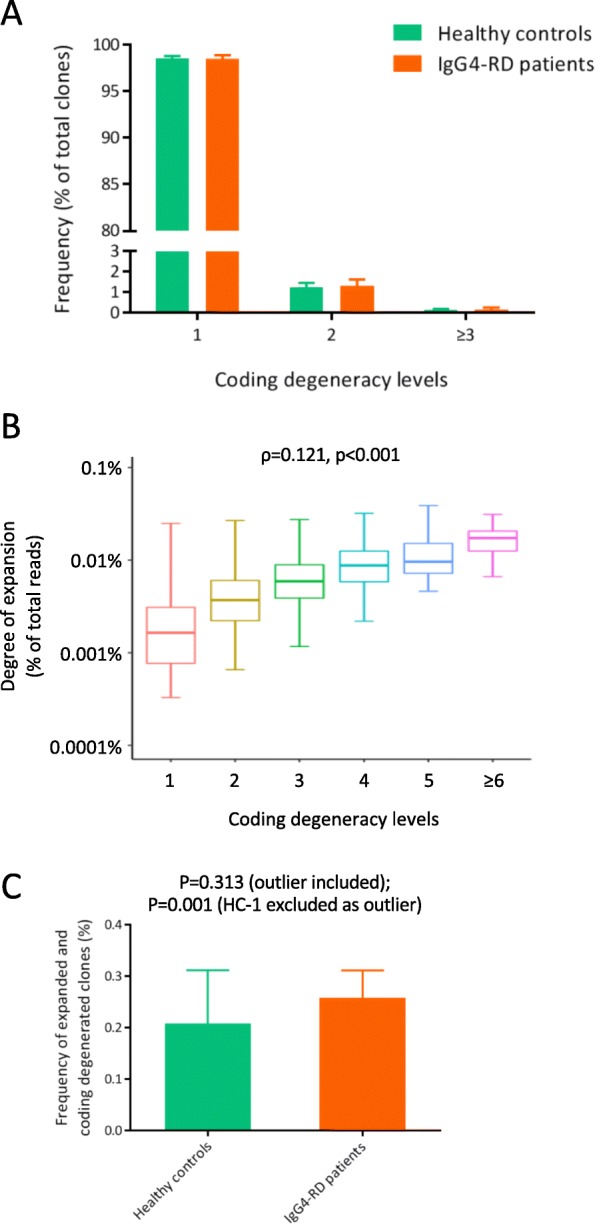
Coding degeneracy levels of CDR3 amino acid clonotypes. The TCR repertoires of all the individuals were downsampled to the same number of reads (158,416 TCR sequences per sample). **a** Distribution of coding degeneracy levels of CDR3 amino acid clonotypes. Data were represented as mean and sd. **b** Boxplot representing frequencies of CDR3 amino acid clonotypes with each distinct coding degeneracy level. The correlation between expansion levels and coding degeneracy levels of TCR clonotypes were analyzed by Spearman rank correlation test. **c** Proportion of expanded (frequency ≥ 0.01%) and coding degenerated (coding degeneracy level ≥ 2) clones in CD4^+^ T cell repertoire from healthy controls and IgG4-RD patients. Data were represented as mean and sd. Difference between the groups was analyzed by Welch’s *t* test

Next, we analyzed the coding degeneracy level of each T cell clonotype, which is a measurement of the number of unique nucleotide sequences encoding a single CDR3 amino acid clonotype, resulting from the degeneracy of genetic code [19]. Here, we also performed analysis on downsampled TCR repertoire data to avoid the bias of sequencing depth. Most of the TCR amino acid clonotypes were encoded by single-nucleotide sequence (Fig. 2a), and the proportion of coding degenerated clones was comparable between healthy controls and IgG4-RD patients (healthy controls, 1.43% ± 0.22%; IgG4-RD patients, 1.5% ± 0.38%; Welch’s *t* test, *p* = 0.684).

Based on the fact that the antigen specificity of TCR is determined by the amino acid sequence, the antigen-driven clonal expansion of T cells should increase the frequency of all the TCR nucleotide clonotypes encoding for the same CDR3 amino acid sequence. Therefore, the expanded and coding degenerated clones are likely driven by antigen-specific expansion, while antigen-nonspecific expansion increases only clonal frequencies, not coding degeneracy levels [19]. We observed a positive correlation between coding degeneracy levels and expansion levels of CDR3 amino acid clonotype, according to the pooled data from all 14 individuals (Fig. 2b), suggesting the existence of antigen-driven clonal expansion in CD4^+^ T cells from peripheral blood. The proportion of expanded (frequency ≥ 0.01%) and coding degenerated (coding degeneracy level ≥ 2) clones was significantly higher in IgG4-RD patients (Fig. 2c) (healthy controls, 0.209% ± 0.103% [outlier included], 0.167% ± 0.022% [HC-1 excluded as outlier]; IgG4-RD patients, 0.259% ± 0.053%; Welch’s *t* test, *p* = 0.313 [outlier included], *p* = 0.001 [outlier excluded]), which suggests the antigen-driven clonal expansion of CD4^+^ T cells in IgG4-RD.

**Fig. 3:**
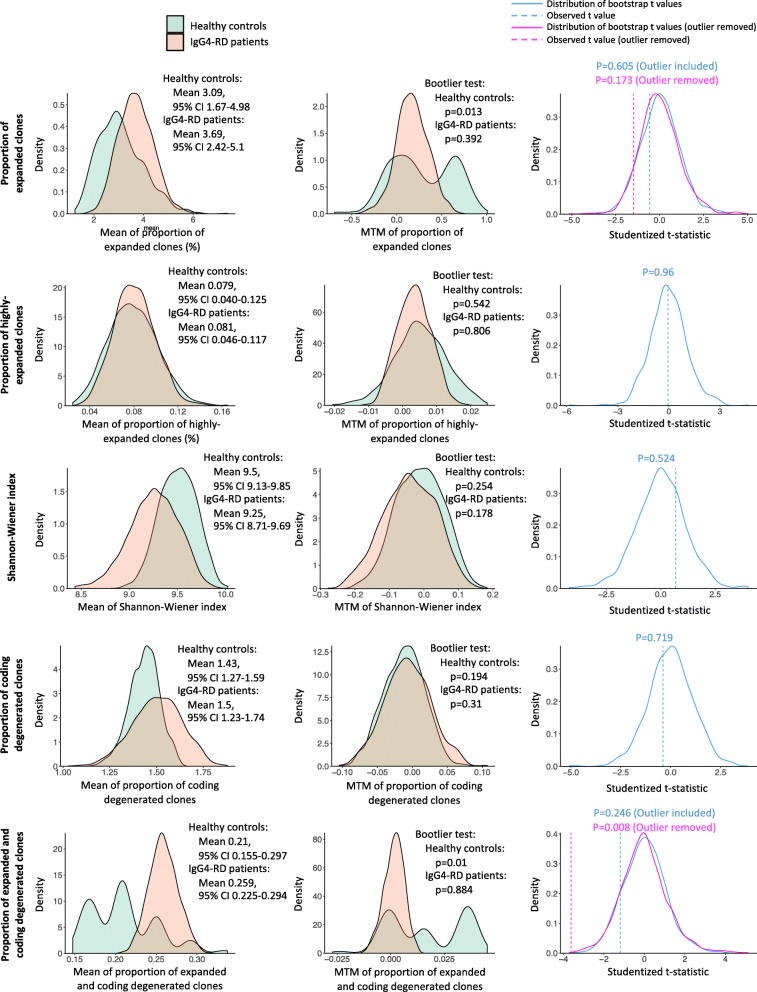
Bootstrap resampling for the parameters associated with clonal expansion and coding degeneracy levels. Bootstrap resampling was applied to the following parameters: the proportion of expanded clones, the proportion of highly expanded clones, the Shannon-Wiener index, the proportion of coding degenerated clones, and the proportion of expanded and coding degenerated clones. Left panel: The distribution of bootstrap means of each variable in healthy controls and IgG4-RD patients. Estimated means and 95% confidence intervals of each variable were also displayed on the plot. Middle panel: Bootlier plot of each variable in healthy controls and IgG4-RD patients. Results of Bootlier test were also displayed on the plot. Right panel: Group comparison by nonparametric bootstrap *t* test with pooled resampling method. Bootstrap *t* values were calculated according to Dwivedi et al. [30]. The distribution of bootstrap *t* values and observed *t* values was shown

Bootstrap resampling allowed us to create a large number of simulated datasets from original samples, to make more credible statistical inference without assumptions about the unknown distribution and to model the sampling distribution under a small sample size. We performed bootstrap resampling for the parameters analyzed above, including the proportion of expanded and highly expanded clones, Shannon-Wiener index, the proportion of coding degenerated clones, and the proportion of expanded and coding degenerated clones. The distribution of bootstrap means, as well as estimated means and 95% confidence intervals of each variable, were shown in the left panel of Fig. 3. The outliers identified above (according to the Dixon criterion and the Grubbs criterion) were consistent with the results of the Bootlier plot and Bootlier test (Fig. 3, middle panel). Group comparison was also performed by a nonparametric bootstrap *t* test with a pooled resampling method, which gave similar results as the previous analysis by Welch’s *t* test (Fig. 3, right panel).

### TRBV and TRBJ gene usage of CD4^+^ T cells in healthy controls and IgG4-RD patients

**Fig. 4:**
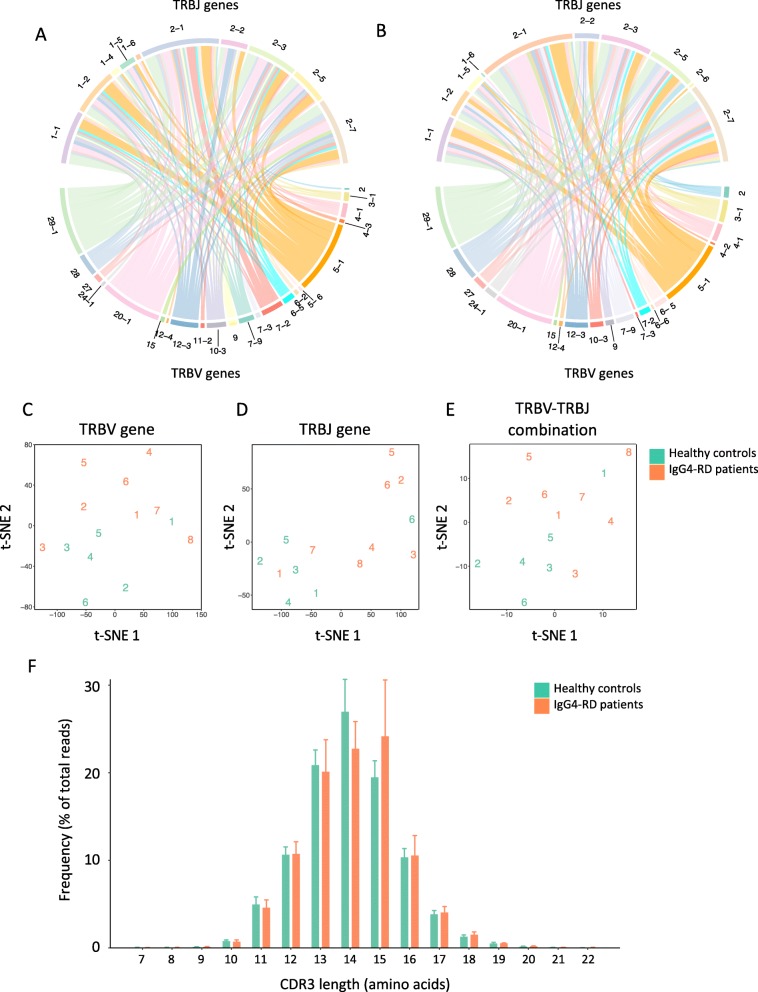
TRBV/TRBJ gene usage and CDR3 length distribution. **a** Circos plot representing the 100 most frequently used TRBV-TRBJ gene combinations in CD4^+^ T cells from healthy controls. **b** Circos plot representing the 100 most frequently used TRBV-TRBJ gene combinations in CD4^+^ T cells from IgG4-RD patients. **c** Visualization of the patterns of TRBV gene usage of each individual by t-SNE dimensionality reduction analysis. **d** Visualization of the patterns of TRBJ gene usage of each individual by t-SNE dimensionality reduction analysis. **e** Visualization of the patterns of TRBV-TRBJ gene combination of each individual by t-SNE dimensionality reduction analysis. **f** CDR3 length distribution of CD4^+^ T cells from healthy controls and IgG4-RD patients. Data were represented as mean and sd

The TRBV and TRBJ gene usage of each clonotype was determined using MiXCR. The 100 most frequently used TRBV-TRBJ combinations in healthy controls and IgG4-RD patients were visualized in Fig. 4a, b. We also visualized the patterns of TRBV and TRBJ gene usage of each individual by t-SNE analysis (Fig. 4c–e), which mapped the multi-dimensional data to a two-dimensional space, preserving pairwise similarities of input objects. According to t-SNE maps, most of the healthy controls had similar patterns of TRBV and TRBJ gene expression, while that of IgG4-RD patients was more heterogeneous. To further study the characteristics of TRBV/TRBJ gene usage in IgG4-RD patients, we compared the expression levels of each TRBV and TRBJ gene and the 100 most frequently used TRBV-TRBJ combinations between healthy controls and IgG4-RD patients (Additional files 4, 5, and 6). However, we did not find any significant difference between healthy controls and IgG4-RD patients after false discovery rate (FDR) control.

### CDR3 length distribution of CD4^+^ T cells in IgG4-RD patients was skewed towards longer fragments

We further investigated the CDR3 length distribution of CD4^+^ T cells from healthy controls and IgG4-RD patients (Fig. 4f). In healthy controls, the length of CDR3 amino acid sequences formed a bell-shaped distribution, which peaks at 14 amino acids. However, the CDR3 length distribution of CD4^+^ T cells from IgG4-RD patients was skewed towards longer sequences and peaks at 15 amino acids. To further compare the CDR3 length distribution, we downsampled the TCR repertoire of each individual to the same number of reads (158,416 TCR sequences per sample) and combined the sequences from the same group together. Analysis by the Mann-Whitney *U* test revealed a significant difference in CDR3 length distribution between healthy controls and IgG4-RD patients (*p* < 0.001).

### Identification of IgG4-RD-specific CDR3 amino acid sequences

**Fig. 5:**
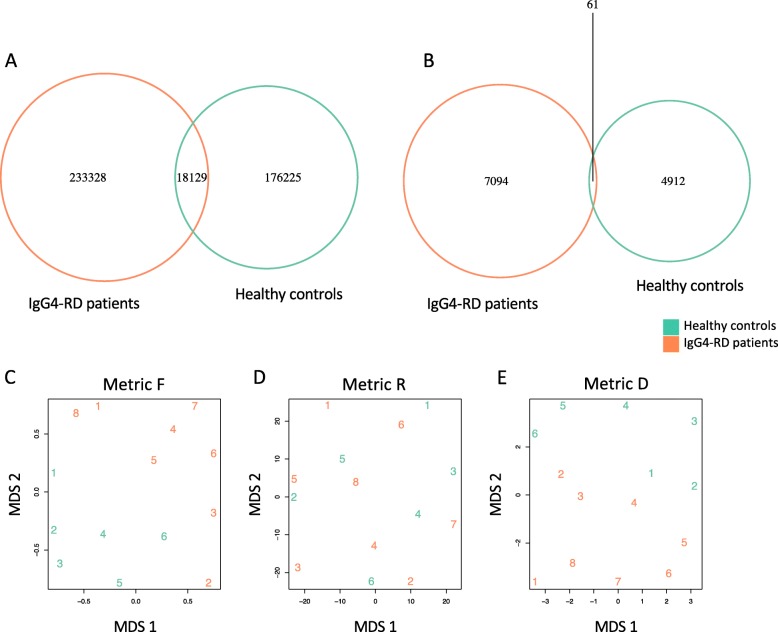
Relative similarity of CD4^+^ T cell repertoires between individuals. **a** Venn diagram showing the overlap of CDR3 amino acid clonotypes identified in healthy controls and IgG4-RD patients. **b** Venn diagram showing the overlap of expanded CDR3 amino acid clonotypes identified in healthy controls and IgG4-RD patients. **c**–**e** Multi-dimensional scaling (MDS) plot in which individuals were projected onto two-dimensional space based on pairwise TCR repertoire similarities. Relative similarity of TCR repertoires were evaluated by metric F (**c**), metric R (**d**), and metric D (**e**). Analysis was performed on downsampled data

Given that it is the amino acid sequence of TCR that determines the structural binding with peptide-MHC complex, the subsequent studies were performed based on the amino acid sequences. A total of 427,682 unique CDR3 amino acid clonotypes of CD4^+^ T cells were identified from the 14 individuals. Among them, 233,328 clonotypes were found in healthy controls, 176,225 clonotypes were found in IgG4-RD patients, and 18,129 clonotypes were shared between healthy controls and IgG4-RD patients (Fig. 5a). As for the expanded clones (frequency ≥ 0.01%), 7094 clonotypes were found in healthy controls, 4912 clonotypes were found in IgG4-RD patients, and 61 clonotypes were found in both (Fig. 5b).

We evaluated the relative similarity of TCR repertoires between individuals by F, R, and D metrics and built an MDS plot for visualization, as described in the “Methods” section. To avoid the bias of sequencing depth, the analysis was performed on downsampled data. According to MDS plots (Fig. 5c–e), TCR repertoires of IgG4-RD patients were separated from that of healthy controls in F and D metrics, while no clear patterns were found in R metrics. Since F and D metrics measure repertoire similarity based on the frequency or number of overlapping sequences, while R metric focuses on the correlation of clonotype frequencies between samples, these data indicated that common clonotypes existed in TCR repertoires among different IgG4-RD patients, but the expansion levels of the shared clonotypes were not correlated in different patients.

To further identify the common TCR clonotypes among IgG4-RD patients, we defined the CDR3 amino acid sequences expanded in at least 2 IgG4-RD patients, while not detected in healthy controls as IgG4-RD-specific sequences, and identified 11 sequences fulfilling these criteria (Table 2). Of note, the sequence “CASSQGTGVRGTEAFF” was identified in 87.5% of IgG4-RD patients but none of the healthy controls and expanded or highly expanded in 3 patients (frequency 6.33%, 0.03%, and 0.02%, respectively).

**Table 2:** IgG4-RD-specific CDR3 amino acid sequences

CDR3 amino acid sequence	IgG4-RD patients with expanded clones	IgG4-RD patients with clones of lower frequencies
CASSSWTGGRYNSPLHF	PT-1, PT-6	
CATSRRPGLEINNEQFF	PT-1, PT-8	
CASSLSGTNEQFF	PT-1, PT-5	
CASSPPGLDFSGANVLTF	PT-2, PT-7	
CASSQGTGVRGTEAFF	PT-4, PT-5, PT-6	PT-1, PT-3, PT-7, PT-9
CSAPTGGNSGANVLTF	PT-4, PT-7	PT-5
CASSLTGTNTEAFF	PT-4, PT-8	PT-1, PT-3, PT-6
CASSFINEQFF	PT-4, PT-8	
CASSLVSSGSNEQFF	PT-4, PT-5	
CAIGQTYEQYF	PT-4, PT-7	
CATSGFGSEQYF	PT-5, PT-6	PT-1, PT-4, PT-7, PT-8

Listed are the CDR3 amino acid sequences expanded in at least two IgG4-RD patients, while not detected in healthy controls (even at lower frequencies)

### Construction of CDR3 similarity networks revealed IgG4-RD-specific clusters

**Fig. 6:**
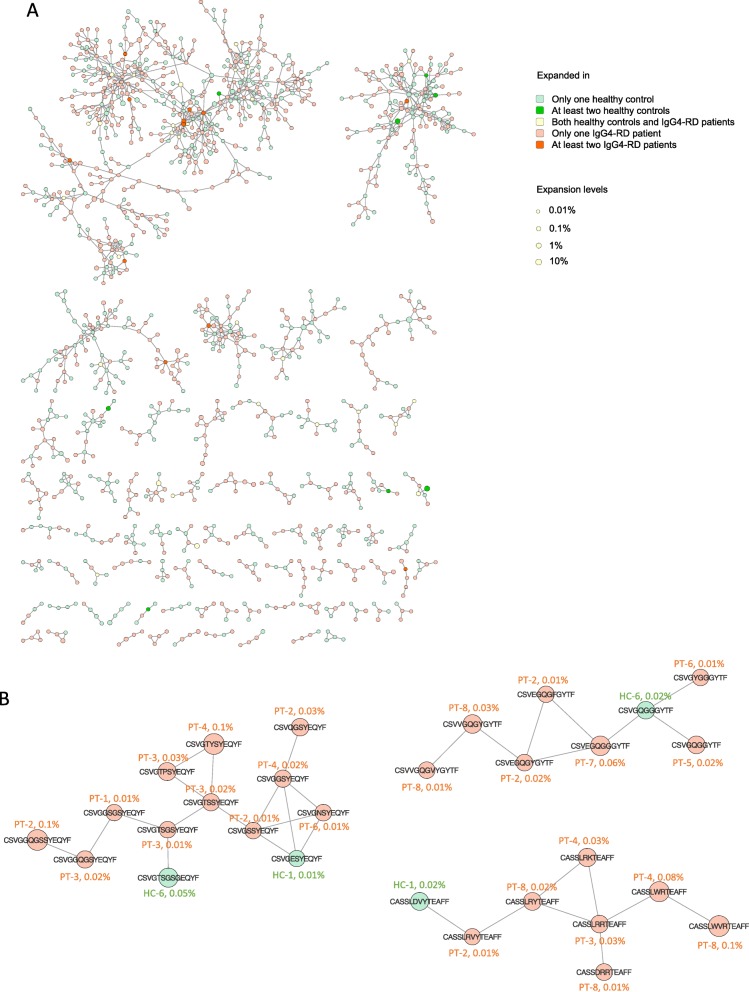
IgG4-RD-specific clusters revealed by CDR3 similarity networks. **a** CDR3 similarity networks constructed by all the CDR3 amino acid clonotypes expanded in at least one individual. Each two CDR3 amino acid clonotypes with a Levenshtein distance of 1 were connected with each other. Node sizes correspond to the expansion levels of the clonotypes. If the clonotypes were expressed by more than one individual, the node sizes were determined by the highest expansion level. **b** IgG4-RD-specific clusters and the CDR3 amino acid sequences, source individuals, and expansion levels of each node in the clusters

The analysis above did not take into consideration the sequence similarity of different CDR3 clonotypes. Thus, we collected all the CDR3 amino acid clonotypes expanded in at least one individual, evaluated the sequence similarity of each two clonotypes by calculating the Levenshtein distance, and constructed CDR3 similarity networks by connecting each two CDR3 clonotypes (nodes) with a Levenshtein distance of 1 [31]. All the clusters comprised at least four connected nodes as shown in Fig. 6a. There were several large CDR3 clusters shared by healthy controls and IgG4-RD patients, probably reflecting the public sequences in the human TCR repertoire [31]. We also identified several IgG4-RD-specific clusters, and the sequences, source individuals, and expansion levels of each node in IgG4-RD-specific clusters were highlighted in Fig. 6b.

### Analysis of TCR sequences with known antigen specificity

**Fig. 7:**
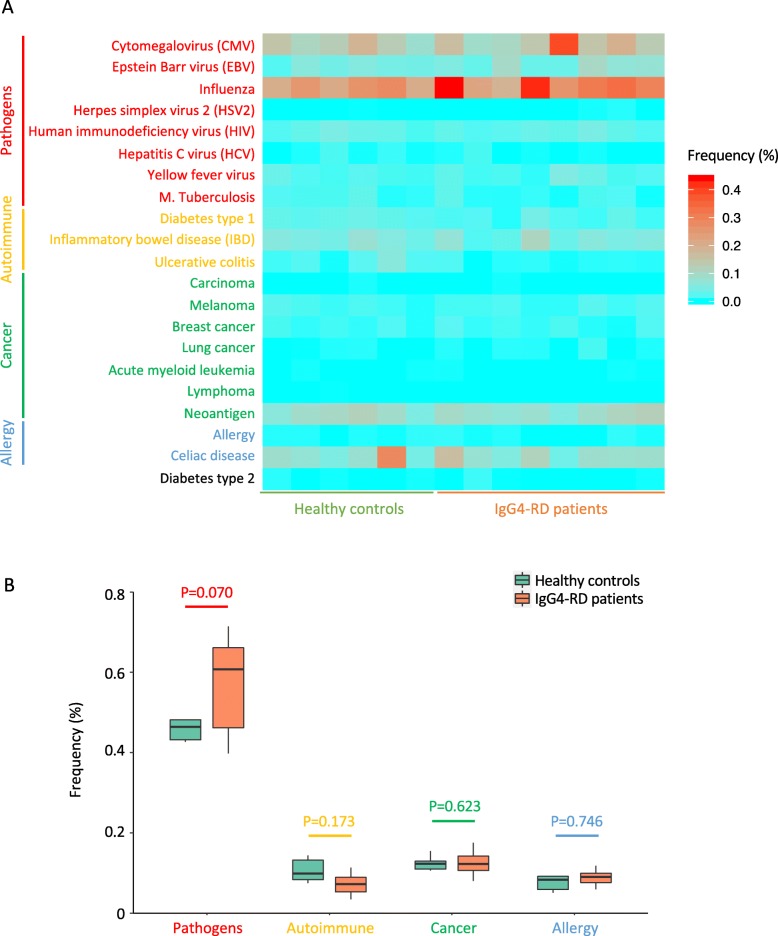
Expression levels of CDR3 sequences associated with known antigen specificity according to McPAS-TCR database. **a** Heatmap presenting the frequencies of sequences associated with each disease in CD4^+^ T cell repertoire of each individual. **b** Boxplot summarizing the frequencies of sequences associated with each immune function in CD4^+^ T cell repertoire of healthy controls and IgG4-RD patients. Difference between the groups was analyzed by Welch’s *t* test (for the frequencies of sequences associated with pathogens, autoimmune, and cancer) or Mann-Whitney *U* test (for the frequencies of sequences associated with allergy). Analysis was performed on downsampled data

In order to reveal the antigen specificity of CD4^+^ T cell clonotypes, we referred to the McPAS-TCR database, which assembled all the TCR sequences with known antigen specificity based on the published literature and classified the immune functions into 4 categories: pathogens, autoimmune, cancer, and allergy [24]. According to the McPAS-TCR database, we collected 9658 annotated human TCR β-chain CDR3 sequences, of which 1091 annotated sequences were present in the CD4^+^ T cell repertoire of our dataset. TCR repertoires were downsampled to 158,416 sequences per sample, and the frequencies of sequences associated with each disease were shown in Fig. 7a. Significant expansion of T cell clonotypes associated with influenza or cytomegalovirus (CMV) was found in 3 IgG4-RD patients. However, after false discovery rate control procedures, no significant difference was identified between healthy controls and IgG4-RD. The expression levels of TCR sequences associated with each immune function were also comparable between healthy controls and IgG4-RD patients (Fig. 7b and Additional file 7).

We also looked up the IgG4-RD-specific sequences identified above (Table 2 and Fig. 6b) in the McPAS database. However, none of them was present in the database.

## Discussion

The pathogenesis of IgG4-RD has been proposed that Tfh cells induce IgG4 class-switching and differentiation of plasmablasts and plasma cells [6, 32], and B cells and plasmablasts present disease-specific antigens and activate CD4^+^ CTLs, which drives the inflammatory and fibrotic processes [3–7, 32]. The critical roles of CD4^+^ T cells in the pathogenesis of IgG4-RD have been demonstrated by this model. Here, using next-generation sequencing, we investigated the TCR repertoire of CD4^+^ T cells from IgG4-RD patients in-depth, in order to reveal the characteristics of immune repertoire in IgG4-RD.

The clonal expansion of CD4^+^ T cells was comparable between healthy controls and IgG4-RD patients, revealed by the proportion of TCR clonotypes in each expansion level, as well as the diversity of TCR repertoire calculated by the Shannon-Wiener index (Fig. 1). However, when we took into consideration the coding degeneracy level of each clone, as an indicator of antigen-driven T cell expansion [19], we found a significantly higher proportion of expanded and coding degenerated clones in CD4^+^ T cells of IgG4-RD patients (Fig. 2), suggesting of antigen-driven clonal expansion in IgG4-RD. Of note, the statistically significant result was based on the removal of the data from HC-1 as an outlier, according to the Dixon criterion, Grubbs criterion, and Bootlier plot. The TCR repertoire is sensitive to environmental stimulation. Therefore, it could be possible that this healthy donor underwent some subclinical infection or hypersensitivity, resulting in antigen-dependent clonal expansion of the TCR repertoire. However, we are also aware of the potential bias caused by outlier removal.

In contrast to our data, Mattoo et al. reported that the CD4^+^ CTLs from the peripheral blood of IgG4-RD patients were oligoclonally expanded [7]. The discrepancy of T cell expansion between these two studies could be possibly explained by the fact that the CD4^+^ CTLs, as a small subpopulation of peripheral CD4^+^ T cells, did not significantly affect the diversity of CD4^+^ T cell pool.

It has been revealed that the patterns of TRBV gene usage were determined dominantly by MHC alleles [33]. Given that the human leukocyte antigen (HLA) complex of IgG4-RD patients is biased towards certain susceptible alleles [34, 35], we hypothesized that the TRBV gene usage of TCR repertoire might also be biased in IgG4-RD. However, we did not find any significant difference in TRBV/TRBJ gene usage between healthy controls and IgG4-RD patients (Additional files 4, 5, and 6), and the t-SNE analysis revealed heterogeneity between patients (Fig. 4c–e).

The distribution of CDR3 length was skewed towards longer fragments in CD4^+^ T cells from IgG4-RD patients (Fig. 4f). A similar pattern of CDR3 length distribution has also been found in peripheral blood T cells from systemic lupus erythematosus patients [36]. It has been reported that antigen exposure resulted in longer CDR3 domains in adults compared to infants in both CD4^+^ and CD8^+^ T cell compartments [37], suggesting that longer CDR3 in IgG4-RD patients might be caused by chronic antigen exposure, which is consistent with the previous results showing antigen-driven clonal expansion in IgG4-RD. However, further study on CDR3 length distribution of TCR is required to fully interpret these data.

TCR repertoire dynamically encodes the antigen exposure history of each individual. TCR repertoire signatures have been identified in a variety of disease, including infectious diseases [38–40], autoimmunity [36, 41], and cancer [42, 43], and could serve as a promising biomarker for diagnosis, prognosis, and monitoring of certain diseases [38, 43, 44]. Here, visualization by MDS analysis revealed repertoire similarities among IgG4-RD patients in F and D metrics (Fig. 5c–e), indicating that disease-specific TCR sequence signatures existed in the CD4^+^ T cell repertoire of IgG4-RD patients, probably driven by common antigens. In addition, we identified the sequence “CASSQGTGVRGTEAFF” (Table 2) that presented in seven of eight IgG4-RD patients but none of the healthy controls. Further study is required to validate the usage of this sequence as a diagnostic biomarker, and to reveal the epitope specificity of this clonotype, thus providing insights for the antigen driving IgG4-RD.

The antigen that triggers IgG4-RD remains unclear. Several antibodies against autoantigens have been identified in IgG4-RD patients, including galectin-3 [45], laminin 511-E8 [46], annexin A11 [47], and prohibitin [48]. However, the positive rate of those antibodies was suboptimal (18~73%), and the antibodies against elements of some organs were undetectable in IgG4-RD patients with other organ involvement [49]. In addition, it is also possible that IgG4-RD could be triggered by the antigens from environmental components, such as pathogens, allergens, and occupational exposure [49–51].

Although of great interest, it is currently impossible to predict the epitopes that T cells recognize based on TCR sequences. However, a recent breakthrough in bioinformatics has allowed us to cluster the TCR sequences with shared epitope specificity [52, 53]. Meysman et al. compared the available approaches of unsupervised TCR clustering based on several different algorithms of CDR3 similarity assessment and reported that compared to more complicated methods, clustering TCR sequences by simply connecting those with a Levenshtein distance of 1 was already of high performance [54]. Here, we constructed TCR networks based on Levenshtein distance and identified several IgG4-RD-specific clusters (Fig. 6), which provides clues for disease-specific antigens.

Another approach of analyzing the antigen specificity of T cell clonotypes is referring to TCR sequence databases [55]. Here, we referred to the McPAS-TCR database, which not only assembled all the TCR sequences with known antigen specificities, but also annotated each sequence according to immune functions. However, no significant difference was found between healthy controls and IgG4-RD patients in the frequency of sequences associated with each disease or immune function (Fig. 7), which could possibly result from the fact that the McPAS database collected only a small fraction of sequences in human TCR repertoire, without the inclusion of IgG4-RD-specific sequences. Of note, although not reaching statistical significance, CD4^+^ T cells from IgG4-RD patients expressed a relatively higher frequency of TCR sequences associated with pathogens (Fig. 7b), suggesting the underlying association between pathogen exposure and IgG4-RD. However, further study is required to confirm this hypothesis.

## Conclusions

In conclusion, using high-throughput sequencing, we analyzed the TCR repertoire of peripheral blood CD4^+^ T cells in IgG4-RD patients in-depth, and found that the TCR repertoire diversity was comparable between healthy controls and IgG4-RD patients, while there was significantly more expanded and coding degenerated clones in CD4^+^ T cell repertoire of IgG4-RD patients, suggesting antigen-driven clonal expansion. The CDR3 length distribution of IgG4-RD patients was skewed towards longer fragments. In addition, IgG4-RD-specific CDR3 sequences and clusters were identified in our study, which provides clues for the disease-specific antigen.

## Supplementary information

**Additional file 1.** : Supplementary methods.

**Additional file 2.** : TCRβ sequence statistics.

**Additional file 3.** : Summary of TCR clonotypes in each sample. (CSV 65491 kb)

**Additional file 4.** : Comparison of TRBV gene usage between healthy controls and IgG4-RD patients.

**Additional file 5.** : Comparison of TRBJ gene usage between healthy controls and IgG4-RD patients.

**Additional file 6.** : Comparison of the 100 most frequently-used TRBV-TRBJ combinations between healthy controls and IgG4-RD patients.

**Additional file 7.** : Bootstrap resampling for the frequency of sequences associated with each immune function. Bootstrap resampling was applied to the following parameters: the frequency of TCR sequences associated with pathogens, autoimmune, cancer, and allergy. Left panel: The distribution of bootstrap means of each variable in healthy controls and IgG4-RD patients. Estimated means and 95% confidence intervals of each variable were also displayed on the plot. Middle panel: Bootlier plot of each variable in healthy controls and IgG4-RD patients. Results of Bootlier test were also displayed on the plot. Right panel: Group comparison by nonparametric bootstrap t-test with pooled resampling method. Bootstrap t values were calculated according to Dwivedi et al. [30]. The distribution of bootstrap t values and observed t values were shown.

## Data Availability

The sequencing data from this study will be made freely available from the NCBI Short Read Archive (SRA).

## Abbreviations

IgG4-RD: IgG4-related disease
5′-RACE: 5′-Rapid amplification of cDNA ends
CDR3: Complementarity determining region 3
Tfh: T follicular helper cell
CTL: Cytotoxic T lymphocytes
TCR: T cell receptor
PBMC: Peripheral blood mononuclear cell
t-SNE: t-distributed stochastic neighbor embedding
MDS: Multi-dimensional scaling
MTM: Mean-trimmed mean
FDR: False discovery rate
CMV: Cytomegalovirus
HLA: Human leukocyte antigen
