# Supplementary material for: High-throughput sequencing of CD4+ T cell repertoire reveals disease-specific signatures in IgG4-related disease
Source: Arthritis Res Ther. 2019 Dec 19;21:295. doi: 10.1186/s13075-019-2069-6 (PMC6923942; doi:10.1186/s13075-019-2069-6)
Supplement: Supplementary file 1 — Additional file 1. : Supplementary methods. [file 13075_2019_2069_MOESM1_ESM.docx]

**Additional file 1: Supplementary methods**

**Bioinformatics analysis**

Shannon-Wiener index was calculated as following:

H’=-$\sum_{i=1}^{s} \frac{\mathrm{ni}}{N}\ln\frac{\mathrm{ni}}{N}$

S is the number of different clones, ni is the number of reads of ith clone, and N is the total number of reads.

TCR repertoire similarities were calculated as following: (1) Metric F: calculated as *F_ij_* = $\sqrt{f_{ij}\times f_{ji}}$, where *f_ij_* is the total frequency of shared clonotypes in sample *i*; (2) Metric R: calculated as *R_ij_* = $\frac{\sum_{k=1}^{n} (f_{ik}-f_{i})(f_{jk}-f_{j})}{\sqrt{\sum_{k=1}^{n} {(f_{ik}-f_{i})}^{2}\sum_{k=1}^{n} {(f_{jk}-f_{j})}^{2}}}$, where *k*=1, 2, …, n, are the indices of shared clonotypes, *f_ik_* is the frequency of *k^th^* clonotype in sample *i*, and *f_i_* is the average frequency of shared clonotypes in sample *i*; (3) Metric D: calculated as *Dij* = $\frac{d_{ij}}{d_{i}\times d_{j}}$, where *d_ij_* is the number of shared clonotypes, and *d_i_* the the number of clonotypes in sample *i*.

IgG4-RD-specific clusters were defined as following: For a cluster of n nodes, in which m nodes were IgG4-RD-specific (expanded in only one IgG4-RD patient, or at least two IgG4-RD patients), we constructed 10,000 random clusters of n nodes based on all the expanded clonotypes. If the occurrence of clusters with ≥m IgG4-RD-specific nodes was <0.1, the cluster was considered as IgG4-RD-specific cluster.

According to McPAS database, we collected all the human TCR β-chain CDR3 sequences, of which the association with antigens were identified by either peptide-MHC multimers or isolation of T cells reactive to specific antigens *in vitro* (antigen identification method = 1 or 2), while the association of TCR sequences with certain pathologies revealed by directly sequencing *ex vivo* T cells (antigen identification method = 3) were excluded. The frequencies of TCR sequences associated with each disease or immune function were calculated as the sum of the counts of TCR sequences associated with each disease/immune function in each sample divided by the total number of TCR sequences in each sample. When we looked up the IgG4-RD-specific sequences and clusters in McPAS database, the sequences of which antigen identification method = 3 were also included.
