# Supplementary material for: High-throughput sequencing of CD4+ T cell repertoire reveals disease-specific signatures in IgG4-related disease
Source: Arthritis Res Ther. 2019 Dec 19;21:295. doi: 10.1186/s13075-019-2069-6 (PMC6923942; doi:10.1186/s13075-019-2069-6)
Supplement: Supplementary file 2 — Additional file 2. : TCRβ sequence statistics. [file 13075_2019_2069_MOESM2_ESM.docx]

**Additional file 2: TCRβ sequence statistics.**

| Individual | Total number of raw reads | Total number of filtered reads | Total number of TCR sequences | Total number of unique clonotypes |
| --- | --- | --- | --- | --- |
| HC-1 | 2020454 | 1394572 | 187284 | 21672 |
| HC-2 | 2228644 | 1727912 | 304637 | 33318 |
| HC-3 | 2219700 | 1663446 | 197645 | 38534 |
| HC-4 | 2310680 | 1737990 | 225370 | 44254 |
| HC-5 | 2165614 | 1599546 | 182498 | 42170 |
| HC-6 | 2124700 | 1527272 | 158416 | 31744 |
| PT-1 | 2261962 | 1785246 | 256643 | 51246 |
| PT-2 | 2160832 | 1589718 | 280798 | 21866 |
| PT-3 | 2259088 | 1652498 | 296847 | 21623 |
| PT-4 | 2252692 | 1664692 | 286297 | 29729 |
| PT-5 | 2256868 | 1608060 | 231124 | 31435 |
| PT-6 | 2149444 | 1602588 | 259725 | 38472 |
| PT-7 | 2236356 | 1703624 | 291031 | 41399 |
| PT-8 | 2168290 | 1681014 | 260133 | 43917 |

Abbreviations: HC: healthy control; PT: IgG4-RD patient.
