# Supplementary material for: High-throughput sequencing of CD4+ T cell repertoire reveals disease-specific signatures in IgG4-related disease
Source: Arthritis Res Ther. 2019 Dec 19;21:295. doi: 10.1186/s13075-019-2069-6 (PMC6923942; doi:10.1186/s13075-019-2069-6)
Supplement: Supplementary file 4 — Additional file 4. : Comparison of TRBV gene usage between healthy controls and IgG4-RD patients. [file 13075_2019_2069_MOESM4_ESM.docx]

**Additional file 4: Comparison of TRBV gene usage between healthy controls and IgG4-RD patients**

| TRBV gene | Frequency in healthy controls (%) | Frequency in IgG4-RD patients (%) | P value^1^ | FDR^2^ | P value^3^  (Bootstrap) | FDR^2^ |
| --- | --- | --- | --- | --- | --- | --- |
| TRBV1 | 0.000678 ± 0.001 | 0.00157 ± 0.00164 | 0.24 | 0.69 | 0.26 | 0.698 |
| TRBV2 | 1.89 ± 0.748 | 3.05 ± 1.93 | 0.181 | 0.69 | 0.159 | 0.698 |
| TRBV3-1 | 2.8 ± 0.885 | 4.43 ± 2.26 | 0.081 | 0.569 | 0.073 | 0.698 |
| TRBV3-2 | 0.00757 ± 0.0085 | 0.00619 ± 0.0116 | 0.535 | 0.822 | 0.798 | 0.876 |
| TRBV4-1 | 3.54 ± 0.992 | 3.82 ± 1.37 | 0.95 | 0.985 | 0.674 | 0.858 |
| TRBV4-2 | 1.45 ± 0.377 | 1.32 ± 0.401 | 0.491 | 0.808 | 0.534 | 0.781 |
| TRBV4-3 | 1.88 ± 1.59 | 0.707 ± 1.02 | 0.491 | 0.808 | 0.152 | 0.698 |
| TRBV5-1 | 13.2 ± 2.27 | 12.7 ± 3.29 | 0.852 | 0.954 | 0.724 | 0.86 |
| TRBV5-3 | 0.00173 ± 0.00184 | 0.000781 ± 0.00106 | 0.314 | 0.69 | 0.283 | 0.698 |
| TRBV5-4 | 0.225 ± 0.023 | 0.272 ± 0.0898 | 0.228 | 0.69 | 0.185 | 0.698 |
| TRBV5-5 | 0.711 ± 0.152 | 0.803 ± 0.236 | 0.345 | 0.69 | 0.396 | 0.744 |
| TRBV5-6 | 1.41 ± 0.633 | 1.25 ± 0.275 | 0.95 | 0.985 | 0.605 | 0.807 |
| TRBV5-7 | 0.00324 ± 0.00289 | 0.00478 ± 0.00417 | 0.518 | 0.822 | 0.415 | 0.744 |
| TRBV5-8 | 0.143 ± 0.0879 | 0.0904 ± 0.0297 | 0.059 | 0.569 | 0.191 | 0.698 |
| TRBV6-1 | 0.743 ± 0.155 | 0.699 ± 0.193 | 0.755 | 0.954 | 0.65 | 0.847 |
| TRBV6-2 | 1.77 ± 0.963 | 1.19 ± 0.812 | 0.282 | 0.69 | 0.263 | 0.698 |
| TRBV6-4 | 0.223 ± 0.0402 | 0.225 ± 0.0843 | 0.852 | 0.954 | 0.966 | 0.966 |
| TRBV6-5 | 3.15 ± 0.736 | 3.24 ± 0.856 | 1 | 1 | 0.813 | 0.876 |
| TRBV6-6 | 1.16 ± 0.202 | 1.27 ± 0.358 | 0.414 | 0.772 | 0.441 | 0.748 |
| TRBV6-7 | 0.00857 ± 0.00577 | 0.00803 ± 0.00891 | 0.573 | 0.822 | 0.903 | 0.942 |
| TRBV6-8 | 0.00991 ± 0.00544 | 0.00639 ± 0.00605 | 0.282 | 0.69 | 0.269 | 0.698 |
| TRBV6-9 | 0.00319 ± 0.00222 | 0.00732 ± 0.00618 | 0.059 | 0.569 | 0.107 | 0.698 |
| TRBV7-2 | 4.74 ± 3.28 | 3.12 ± 1.34 | 0.282 | 0.69 | 0.299 | 0.698 |
| TRBV7-3 | 1.58 ± 0.697 | 1.31 ± 0.306 | 0.852 | 0.954 | 0.413 | 0.744 |
| TRBV7-4 | 0.00583 ± 0.00402 | 0.00787 ± 0.00727 | 0.755 | 0.954 | 0.519 | 0.781 |
| TRBV7-6 | 0.551 ± 0.168 | 0.663 ± 0.349 | 0.662 | 0.904 | 0.489 | 0.761 |
| TRBV7-7 | 0.141 ± 0.0315 | 0.15 ± 0.0582 | 0.662 | 0.904 | 0.737 | 0.86 |
| TRBV7-8 | 0.729 ± 0.264 | 0.89 ± 0.241 | 0.282 | 0.69 | 0.275 | 0.698 |
| TRBV7-9 | 3.26 ± 0.576 | 3.78 ± 0.859 | 0.081 | 0.569 | 0.19 | 0.698 |
| TRBV9 | 2.63 ± 0.81 | 2.49 ± 0.424 | 0.852 | 0.954 | 0.711 | 0.86 |
| TRBV10-1 | 0.417 ± 0.199 | 0.202 ± 0.133 | 0.043 | 0.569 | 0.031 | 0.434 |
| TRBV10-2 | 0.58 ± 0.107 | 0.497 ± 0.15 | 0.345 | 0.69 | 0.237 | 0.698 |
| TRBV10-3 | 3.99 ± 1.15 | 3.29 ± 1.52 | 0.282 | 0.69 | 0.34 | 0.744 |
| TRBV11-1 | 0.235 ± 0.0842 | 0.197 ± 0.061 | 0.852 | 0.954 | 0.402 | 0.744 |
| TRBV11-2 | 1.49 ± 0.474 | 1.26 ± 0.358 | 0.573 | 0.822 | 0.351 | 0.744 |
| TRBV11-3 | 0.302 ± 0.495 | 0.133 ± 0.0739 | 0.755 | 0.954 | 0.572 | 0.781 |
| TRBV12-2 | 8.43e-05 ± 0.000207 | 0 ± 0 | 0.312 | 0.69 | 0.38618524 | 0.744 |
| TRBV12-3 | 5.52 ± 1.92 | 4.52 ± 1.17 | 0.282 | 0.69 | 0.288 | 0.698 |
| TRBV12-4 | 1.97 ± 0.565 | 2.01 ± 0.757 | 0.852 | 0.954 | 0.908 | 0.942 |
| TRBV12-5 | 0.0765 ± 0.0238 | 0.105 ± 0.0443 | 0.491 | 0.808 | 0.146 | 0.698 |
| TRBV13 | 0.154 ± 0.273 | 0.056 ± 0.0362 | 1 | 1 | 0.557 | 0.781 |
| TRBV14 | 0.242 ± 0.0944 | 0.26 ± 0.124 | 0.95 | 0.985 | 0.771 | 0.87 |
| TRBV15 | 2.24 ± 0.868 | 1.74 ± 0.729 | 0.142 | 0.69 | 0.292 | 0.698 |
| TRBV16 | 0.0333 ± 0.00961 | 0.0293 ± 0.0164 | 0.491 | 0.808 | 0.566 | 0.781 |
| TRBV18 | 0.372 ± 0.0562 | 0.587 ± 0.156 | 0.013 | 0.559 | 0.005 | 0.28 |
| TRBV19 | 0.148 ± 0.0543 | 0.174 ± 0.0798 | 0.573 | 0.822 | 0.483 | 0.761 |
| TRBV20-1 | 11.4 ± 2 | 11.1 ± 6.54 | 0.228 | 0.69 | 0.933 | 0.95 |
| TRBV21-1 | 0.372 ± 0.0773 | 0.413 ± 0.117 | 0.414 | 0.772 | 0.425 | 0.744 |
| TRBV23-1 | 0.00444 ± 0.00237 | 0.00982 ± 0.00508 | 0.081 | 0.569 | 0.019 | 0.355 |
| TRBV24 | 0.0918 ± 0.141 | 0.0597 ± 0.166 | 0.322 | 0.69 | 0.726 | 0.86 |
| TRBV24-1 | 1.71 ± 0.564 | 2.91 ± 0.863 | 0.02 | 0.559 | 0.011 | 0.308 |
| TRBV25-1 | 0.68 ± 0.201 | 0.56 ± 0.105 | 0.282 | 0.69 | 0.222 | 0.698 |
| TRBV27 | 2.54 ± 0.663 | 3.29 ± 1 | 0.228 | 0.69 | 0.121 | 0.698 |
| TRBV28 | 4.61 ± 1.45 | 6.55 ± 3.15 | 0.108 | 0.671 | 0.172 | 0.698 |
| TRBV29-1 | 12.7 ± 2.52 | 12.3 ± 1.74 | 0.95 | 0.985 | 0.777 | 0.87 |
| TRBV30 | 0.184 ± 0.0861 | 0.154 ± 0.0661 | 0.345 | 0.69 | 0.486 | 0.761 |

^1^P-values were calculated by Mann-Whitney U test.

^2^False discovery rate (FDR) control was performed by Benjamini-Hochberg procedure.

^3^P-values were calculated by nonparametric bootstrap t-test with pooled resampling method.
