# Supplementary material for: High-throughput sequencing of CD4+ T cell repertoire reveals disease-specific signatures in IgG4-related disease
Source: Arthritis Res Ther. 2019 Dec 19;21:295. doi: 10.1186/s13075-019-2069-6 (PMC6923942; doi:10.1186/s13075-019-2069-6)
Supplement: Supplementary file 5 — Additional file 5. : Comparison of TRBJ gene usage between healthy controls and IgG4-RD patients. [file 13075_2019_2069_MOESM5_ESM.docx]

**Additional file 5: Comparison of TRBJ gene usage between healthy controls and IgG4-RD patients**

| TRBJ gene | Frequency in healthy controls (%) | Frequency in IgG4-RD patients (%) | P value^1^ | FDR^2^ | P value^3^  (Bootstrap) | FDR^2^ |
| --- | --- | --- | --- | --- | --- | --- |
| TRBJ1-1 | 12.2 ± 2.64 | 10.7 ± 2.38 | 0.414 | 0.597 | 0.303 | 0.438 |
| TRBJ1-2 | 10.5 ± 3.19 | 7.71 ± 1.8 | 0.059 | 0.281 | 0.072 | 0.237 |
| TRBJ1-3 | 2.26 ± 0.402 | 1.84 ± 0.345 | 0.081 | 0.281 | 0.066 | 0.237 |
| TRBJ1-4 | 3.07 ± 0.95 | 2.27 ± 0.568 | 0.108 | 0.281 | 0.091 | 0.237 |
| TRBJ1-5 | 5.93 ± 0.644 | 5.62 ± 1.08 | 0.755 | 0.817 | 0.511 | 0.664 |
| TRBJ1-6 | 3.22 ± 0.741 | 2.46 ± 0.817 | 0.228 | 0.459 | 0.083 | 0.237 |
| TRBJ2-1 | 15.5 ± 3.43 | 19.3 ± 5.71 | 0.059 | 0.281 | 0.131 | 0.284 |
| TRBJ2-2 | 7.7 ± 2.56 | 7.2 ± 0.979 | 0.573 | 0.745 | 0.69 | 0.747 |
| TRBJ2-3 | 11.6 ± 0.986 | 11.3 ± 1.31 | 0.662 | 0.782 | 0.613 | 0.724 |
| TRBJ2-4 | 1.88 ± 0.378 | 1.83 ± 0.362 | 0.95 | 0.95 | 0.787 | 0.787 |
| TRBJ2-5 | 9.87 ± 0.844 | 11 ± 2.6 | 0.282 | 0.459 | 0.268 | 0.436 |
| TRBJ2-6 | 1.81 ± 0.299 | 2.03 ± 0.384 | 0.282 | 0.459 | 0.256 | 0.436 |
| TRBJ2-7 | 14.4 ± 1.79 | 16.6 ± 1.93 | 0.108 | 0.281 | 0.045 | 0.237 |

^1^P-values were calculated by Mann-Whitney U test.

^2^False discovery rate (FDR) control was performed by Benjamini-Hochberg procedure.

^3^P-values were calculated by nonparametric bootstrap t-test with pooled resampling method.
