# Supplementary material for: High-throughput sequencing of CD4+ T cell repertoire reveals disease-specific signatures in IgG4-related disease
Source: Arthritis Res Ther. 2019 Dec 19;21:295. doi: 10.1186/s13075-019-2069-6 (PMC6923942; doi:10.1186/s13075-019-2069-6)
Supplement: Supplementary file 6 — Additional file 6. : Comparison of the 100 most frequently-used TRBV-TRBJ combinations between healthy controls and IgG4-RD patients. [file 13075_2019_2069_MOESM6_ESM.docx]

**Additional file 6: Comparison of the 100 most frequently-used TRBV-TRBJ combinations** **between healthy controls and IgG4-RD patients**

| TRBV/TRBJ combination | Frequency in healthy controls (%) | Frequency in IgG4-RD patients (%) | P value^1^ | FDR^2^ | P value^3^  (Bootstrap) | FDR^2^ |
| --- | --- | --- | --- | --- | --- | --- |
| TRBV3-1/TRBJ1-1 | 0.375 ± 0.222 | 1.18 ± 2.22 | 0.345 | 0.639 | 0.522 | 0.737 |
| TRBV4-1/TRBJ1-1 | 0.347 ± 0.119 | 0.291 ± 0.119 | 0.491 | 0.711 | 0.407 | 0.683 |
| TRBV5-1/TRBJ1-1 | 1.84 ± 0.859 | 1.66 ± 1.5 | 0.181 | 0.49 | 0.816 | 0.907 |
| TRBV6-5/TRBJ1-1 | 0.458 ± 0.133 | 0.672 ± 0.899 | 0.414 | 0.656 | 0.71 | 0.855 |
| TRBV7-9/TRBJ1-1 | 0.425 ± 0.119 | 0.421 ± 0.113 | 0.852 | 0.936 | 0.941 | 0.958 |
| TRBV10-3/TRBJ1-1 | 0.781 ± 0.817 | 0.302 ± 0.14 | 0.043 | 0.328 | 0.081 | 0.562 |
| TRBV12-3/TRBJ1-1 | 0.644 ± 0.233 | 0.612 ± 0.303 | 0.852 | 0.936 | 0.812 | 0.907 |
| TRBV20-1/TRBJ1-1 | 1.19 ± 0.252 | 0.773 ± 0.212 | 0.013 | 0.25 | 0.007 | 0.267 |
| TRBV27/TRBJ1-1 | 0.308 ± 0.0818 | 0.323 ± 0.217 | 0.662 | 0.871 | 0.891 | 0.948 |
| TRBV28/TRBJ1-1 | 0.395 ± 0.123 | 0.457 ± 0.192 | 0.414 | 0.656 | 0.53 | 0.737 |
| TRBV29-1/TRBJ1-1 | 1.84 ± 0.401 | 1.35 ± 0.33 | 0.043 | 0.328 | 0.034 | 0.34 |
| TRBV3-1/TRBJ1-2 | 0.295 ± 0.124 | 0.316 ± 0.126 | 0.852 | 0.936 | 0.745 | 0.887 |
| TRBV4-1/TRBJ1-2 | 0.373 ± 0.146 | 0.435 ± 0.295 | 0.95 | 0.979 | 0.641 | 0.811 |
| TRBV5-1/TRBJ1-2 | 2.18 ± 3.17 | 0.758 ± 0.231 | 0.228 | 0.531 | 0.202 | 0.587 |
| TRBV6-5/TRBJ1-2 | 0.437 ± 0.14 | 0.33 ± 0.128 | 0.108 | 0.45 | 0.161 | 0.587 |
| TRBV7-9/TRBJ1-2 | 0.354 ± 0.102 | 0.34 ± 0.0915 | 0.95 | 0.979 | 0.771 | 0.897 |
| TRBV10-3/TRBJ1-2 | 0.353 ± 0.112 | 0.247 ± 0.152 | 0.282 | 0.588 | 0.222 | 0.587 |
| TRBV12-3/TRBJ1-2 | 0.55 ± 0.205 | 0.48 ± 0.269 | 0.414 | 0.656 | 0.597 | 0.765 |
| TRBV20-1/TRBJ1-2 | 1.01 ± 0.118 | 0.637 ± 0.204 | 0.001 | 0.067 | 0.003 | 0.267 |
| TRBV28/TRBJ1-2 | 0.491 ± 0.119 | 0.581 ± 0.25 | 0.345 | 0.639 | 0.434 | 0.694 |
| TRBV29-1/TRBJ1-2 | 1.4 ± 0.353 | 1.08 ± 0.452 | 0.142 | 0.489 | 0.167 | 0.587 |
| TRBV5-1/TRBJ1-4 | 0.374 ± 0.162 | 0.253 ± 0.115 | 0.142 | 0.489 | 0.108 | 0.562 |
| TRBV5-1/TRBJ1-5 | 0.627 ± 0.684 | 0.365 ± 0.0882 | 0.662 | 0.871 | 0.355 | 0.668 |
| TRBV12-3/TRBJ1-5 | 0.38 ± 0.123 | 0.388 ± 0.135 | 1 | 1 | 0.919 | 0.958 |
| TRBV20-1/TRBJ1-5 | 0.904 ± 0.291 | 0.665 ± 0.181 | 0.108 | 0.45 | 0.072 | 0.554 |
| TRBV28/TRBJ1-5 | 0.341 ± 0.113 | 0.611 ± 0.541 | 0.081 | 0.387 | 0.283 | 0.594 |
| TRBV29-1/TRBJ1-5 | 0.493 ± 0.201 | 0.384 ± 0.0851 | 0.414 | 0.656 | 0.21 | 0.587 |
| TRBV5-1/TRBJ1-6 | 0.346 ± 0.131 | 0.242 ± 0.092 | 0.142 | 0.489 | 0.1 | 0.562 |
| TRBV20-1/TRBJ1-6 | 0.416 ± 0.164 | 0.264 ± 0.106 | 0.059 | 0.37 | 0.054 | 0.491 |
| TRBV2/TRBJ2-1 | 0.279 ± 0.113 | 0.346 ± 0.131 | 0.414 | 0.656 | 0.334 | 0.665 |
| TRBV3-1/TRBJ2-1 | 0.451 ± 0.234 | 0.572 ± 0.166 | 0.181 | 0.49 | 0.282 | 0.594 |
| TRBV4-1/TRBJ2-1 | 0.451 ± 0.216 | 0.661 ± 0.347 | 0.282 | 0.588 | 0.211 | 0.587 |
| TRBV5-1/TRBJ2-1 | 1.66 ± 0.326 | 2.75 ± 1.39 | 0.043 | 0.328 | 0.06 | 0.5 |
| TRBV5-6/TRBJ2-1 | 0.409 ± 0.487 | 0.208 ± 0.0762 | 0.573 | 0.796 | 0.268 | 0.594 |
| TRBV6-5/TRBJ2-1 | 0.358 ± 0.101 | 0.328 ± 0.0689 | 0.662 | 0.871 | 0.529 | 0.737 |
| TRBV7-2/TRBJ2-1 | 1.61 ± 2.68 | 0.513 ± 0.196 | 0.414 | 0.656 | 0.269 | 0.594 |
| TRBV7-3/TRBJ2-1 | 0.378 ± 0.11 | 0.342 ± 0.0871 | 0.662 | 0.871 | 0.518 | 0.737 |
| TRBV7-9/TRBJ2-1 | 0.389 ± 0.0703 | 0.709 ± 0.281 | 0.008 | 0.2 | 0.014 | 0.28 |
| TRBV9/TRBJ2-1 | 0.413 ± 0.123 | 0.515 ± 0.144 | 0.282 | 0.588 | 0.19 | 0.587 |
| TRBV10-3/TRBJ2-1 | 0.49 ± 0.0505 | 0.526 ± 0.15 | 1 | 1 | 0.584 | 0.765 |
| TRBV11-2/TRBJ2-1 | 0.3 ± 0.162 | 0.26 ± 0.0745 | 0.755 | 0.877 | 0.564 | 0.752 |
| TRBV12-3/TRBJ2-1 | 0.645 ± 0.197 | 0.789 ± 0.325 | 0.491 | 0.711 | 0.361 | 0.668 |
| TRBV12-4/TRBJ2-1 | 0.322 ± 0.21 | 0.329 ± 0.19 | 0.755 | 0.877 | 0.951 | 0.958 |
| TRBV15/TRBJ2-1 | 0.229 ± 0.04 | 0.488 ± 0.713 | 0.345 | 0.639 | 0.524 | 0.737 |
| TRBV20-1/TRBJ2-1 | 1.58 ± 0.465 | 4.04 ± 7.08 | 0.755 | 0.877 | 0.552 | 0.746 |
| TRBV24-1/TRBJ2-1 | 0.457 ± 0.15 | 0.705 ± 0.186 | 0.029 | 0.326 | 0.021 | 0.3 |
| TRBV27/TRBJ2-1 | 0.283 ± 0.0812 | 0.403 ± 0.061 | 0.02 | 0.25 | 0.011 | 0.275 |
| TRBV28/TRBJ2-1 | 0.539 ± 0.237 | 0.75 ± 0.34 | 0.142 | 0.489 | 0.223 | 0.587 |
| TRBV29-1/TRBJ2-1 | 2.01 ± 0.513 | 2.12 ± 0.504 | 0.755 | 0.877 | 0.682 | 0.849 |
| TRBV3-1/TRBJ2-2 | 0.202 ± 0.104 | 0.578 ± 0.786 | 0.059 | 0.37 | 0.282 | 0.594 |
| TRBV5-1/TRBJ2-2 | 0.904 ± 0.189 | 1.04 ± 0.295 | 0.181 | 0.49 | 0.37 | 0.668 |
| TRBV7-3/TRBJ2-2 | 0.386 ± 0.586 | 0.193 ± 0.0652 | 0.491 | 0.711 | 0.455 | 0.7 |
| TRBV9/TRBJ2-2 | 0.319 ± 0.194 | 0.269 ± 0.0533 | 0.95 | 0.979 | 0.538 | 0.737 |
| TRBV10-3/TRBJ2-2 | 0.379 ± 0.25 | 0.247 ± 0.147 | 0.282 | 0.588 | 0.278 | 0.594 |
| TRBV12-3/TRBJ2-2 | 1.22 ± 2.15 | 0.327 ± 0.0983 | 0.491 | 0.711 | 0.251 | 0.594 |
| TRBV20-1/TRBJ2-2 | 0.503 ± 0.0777 | 0.416 ± 0.15 | 0.228 | 0.531 | 0.223 | 0.587 |
| TRBV28/TRBJ2-2 | 0.225 ± 0.0931 | 0.324 ± 0.138 | 0.081 | 0.387 | 0.15 | 0.587 |
| TRBV29-1/TRBJ2-2 | 0.886 ± 0.29 | 0.789 ± 0.183 | 0.755 | 0.877 | 0.451 | 0.7 |
| TRBV3-1/TRBJ2-3 | 0.274 ± 0.103 | 0.283 ± 0.0797 | 0.414 | 0.656 | 0.864 | 0.939 |
| TRBV4-1/TRBJ2-3 | 0.35 ± 0.116 | 0.414 ± 0.106 | 0.228 | 0.531 | 0.297 | 0.606 |
| TRBV5-1/TRBJ2-3 | 1.45 ± 0.314 | 1.42 ± 0.289 | 0.852 | 0.936 | 0.878 | 0.944 |
| TRBV6-5/TRBJ2-3 | 0.292 ± 0.0966 | 0.277 ± 0.104 | 1 | 1 | 0.79 | 0.907 |
| TRBV7-2/TRBJ2-3 | 0.464 ± 0.197 | 0.43 ± 0.206 | 0.755 | 0.877 | 0.771 | 0.897 |
| TRBV7-9/TRBJ2-3 | 0.399 ± 0.152 | 0.404 ± 0.11 | 0.755 | 0.877 | 0.947 | 0.958 |
| TRBV9/TRBJ2-3 | 0.386 ± 0.0497 | 0.339 ± 0.0772 | 0.181 | 0.49 | 0.222 | 0.587 |
| TRBV10-3/TRBJ2-3 | 0.405 ± 0.0959 | 0.4 ± 0.195 | 0.852 | 0.936 | 0.958 | 0.958 |
| TRBV12-3/TRBJ2-3 | 0.427 ± 0.138 | 0.389 ± 0.125 | 0.491 | 0.711 | 0.589 | 0.765 |
| TRBV20-1/TRBJ2-3 | 1.77 ± 0.682 | 1.13 ± 0.275 | 0.108 | 0.45 | 0.027 | 0.3 |
| TRBV24-1/TRBJ2-3 | 0.254 ± 0.0748 | 0.444 ± 0.259 | 0.02 | 0.25 | 0.102 | 0.562 |
| TRBV27/TRBJ2-3 | 0.242 ± 0.076 | 0.643 ± 0.849 | 0.059 | 0.37 | 0.271 | 0.594 |
| TRBV28/TRBJ2-3 | 0.424 ± 0.183 | 0.615 ± 0.373 | 0.228 | 0.531 | 0.285 | 0.594 |
| TRBV29-1/TRBJ2-3 | 1.75 ± 0.459 | 1.7 ± 0.47 | 0.95 | 0.979 | 0.832 | 0.914 |
| TRBV2/TRBJ2-5 | 0.185 ± 0.0668 | 0.942 ± 1.9 | 0.181 | 0.49 | 0.437 | 0.694 |
| TRBV4-1/TRBJ2-5 | 0.351 ± 0.073 | 0.304 ± 0.115 | 0.414 | 0.656 | 0.406 | 0.683 |
| TRBV5-1/TRBJ2-5 | 1.51 ± 0.267 | 1.5 ± 0.427 | 0.95 | 0.979 | 0.948 | 0.958 |
| TRBV7-2/TRBJ2-5 | 0.413 ± 0.199 | 0.371 ± 0.189 | 0.755 | 0.877 | 0.694 | 0.849 |
| TRBV7-9/TRBJ2-5 | 0.277 ± 0.0551 | 0.387 ± 0.165 | 0.081 | 0.387 | 0.141 | 0.587 |
| TRBV10-3/TRBJ2-5 | 0.327 ± 0.0977 | 0.385 ± 0.191 | 0.755 | 0.877 | 0.489 | 0.737 |
| TRBV12-3/TRBJ2-5 | 0.369 ± 0.11 | 0.354 ± 0.119 | 0.755 | 0.877 | 0.8 | 0.907 |
| TRBV20-1/TRBJ2-5 | 1.17 ± 0.303 | 0.879 ± 0.35 | 0.081 | 0.387 | 0.118 | 0.562 |
| TRBV24-1/TRBJ2-5 | 0.195 ± 0.08 | 0.35 ± 0.362 | 0.345 | 0.639 | 0.381 | 0.668 |
| TRBV28/TRBJ2-5 | 0.419 ± 0.149 | 1.11 ± 1.68 | 0.282 | 0.588 | 0.41 | 0.683 |
| TRBV29-1/TRBJ2-5 | 1.44 ± 0.25 | 1.57 ± 0.349 | 0.491 | 0.711 | 0.428 | 0.694 |
| TRBV5-1/TRBJ2-6 | 0.243 ± 0.0989 | 0.314 ± 0.104 | 0.181 | 0.49 | 0.214 | 0.587 |
| TRBV2/TRBJ2-7 | 0.227 ± 0.0878 | 0.345 ± 0.115 | 0.081 | 0.387 | 0.087 | 0.562 |
| TRBV3-1/TRBJ2-7 | 0.404 ± 0.132 | 0.678 ± 0.198 | 0.005 | 0.2 | 0.008 | 0.267 |
| TRBV4-1/TRBJ2-7 | 0.55 ± 0.0987 | 0.701 ± 0.285 | 0.181 | 0.49 | 0.267 | 0.594 |
| TRBV5-1/TRBJ2-7 | 1.72 ± 0.348 | 2.07 ± 0.506 | 0.345 | 0.639 | 0.167 | 0.587 |
| TRBV6-5/TRBJ2-7 | 0.445 ± 0.112 | 0.532 ± 0.114 | 0.345 | 0.639 | 0.189 | 0.587 |
| TRBV7-2/TRBJ2-7 | 0.718 ± 0.373 | 0.546 ± 0.329 | 0.573 | 0.796 | 0.376 | 0.668 |
| TRBV7-9/TRBJ2-7 | 0.453 ± 0.083 | 0.63 ± 0.161 | 0.043 | 0.328 | 0.027 | 0.3 |
| TRBV9/TRBJ2-7 | 0.264 ± 0.0617 | 0.339 ± 0.114 | 0.228 | 0.531 | 0.2 | 0.587 |
| TRBV10-3/TRBJ2-7 | 0.6 ± 0.185 | 0.655 ± 0.28 | 0.95 | 0.979 | 0.696 | 0.849 |
| TRBV12-3/TRBJ2-7 | 0.57 ± 0.13 | 0.673 ± 0.125 | 0.228 | 0.531 | 0.154 | 0.587 |
| TRBV15/TRBJ2-7 | 0.657 ± 0.825 | 0.229 ± 0.0692 | 0.142 | 0.489 | 0.112 | 0.562 |
| TRBV20-1/TRBJ2-7 | 1.85 ± 0.529 | 1.61 ± 0.381 | 0.573 | 0.796 | 0.345 | 0.665 |
| TRBV24-1/TRBJ2-7 | 0.138 ± 0.0551 | 0.588 ± 1 | 0.008 | 0.2 | 0.346 | 0.665 |
| TRBV27/TRBJ2-7 | 0.446 ± 0.0804 | 0.582 ± 0.104 | 0.02 | 0.25 | 0.02 | 0.3 |
| TRBV28/TRBJ2-7 | 1.15 ± 0.474 | 1.33 ± 0.594 | 0.414 | 0.656 | 0.537 | 0.737 |
| TRBV29-1/TRBJ2-7 | 1.76 ± 0.434 | 2.13 ± 0.354 | 0.181 | 0.49 | 0.114 | 0.562 |

^1^P-values were calculated by Mann-Whitney U test.

^2^False discovery rate (FDR) control was performed by Benjamini-Hochberg procedure.

^3^P-values were calculated by nonparametric bootstrap t-test with pooled resampling method.
