# Supplementary material for: High-throughput sequencing of CD4+ T cell repertoire reveals disease-specific signatures in IgG4-related disease
Source: Arthritis Res Ther. 2019 Dec 19;21:295. doi: 10.1186/s13075-019-2069-6 (PMC6923942; doi:10.1186/s13075-019-2069-6)
Supplement: Supplementary file 7 — Additional file 7. : Bootstrap resampling for the frequency of sequences associated with each immune function. Bootstrap resampling was applied to the following parameters: the frequency of TCR sequences associated with pathogens, autoimmune, cancer, and allergy. Left panel: The distribution of bootstrap means of each variable in healthy controls and IgG4-RD patients. Estimated means and 95% confidence intervals of each variable were also displayed on the plot. Middle panel: Bootlier plot of each variable in healthy controls and IgG4-RD patients. Results of Bootlier test were also displayed on the plot. Right panel: Group comparison by nonparametric bootstrap t-test with pooled resampling method. Bootstrap t values were calculated according to Dwivedi et al. [30]. The distribution of bootstrap t values and observed t values were shown. [file 13075_2019_2069_MOESM7_ESM.pdf]

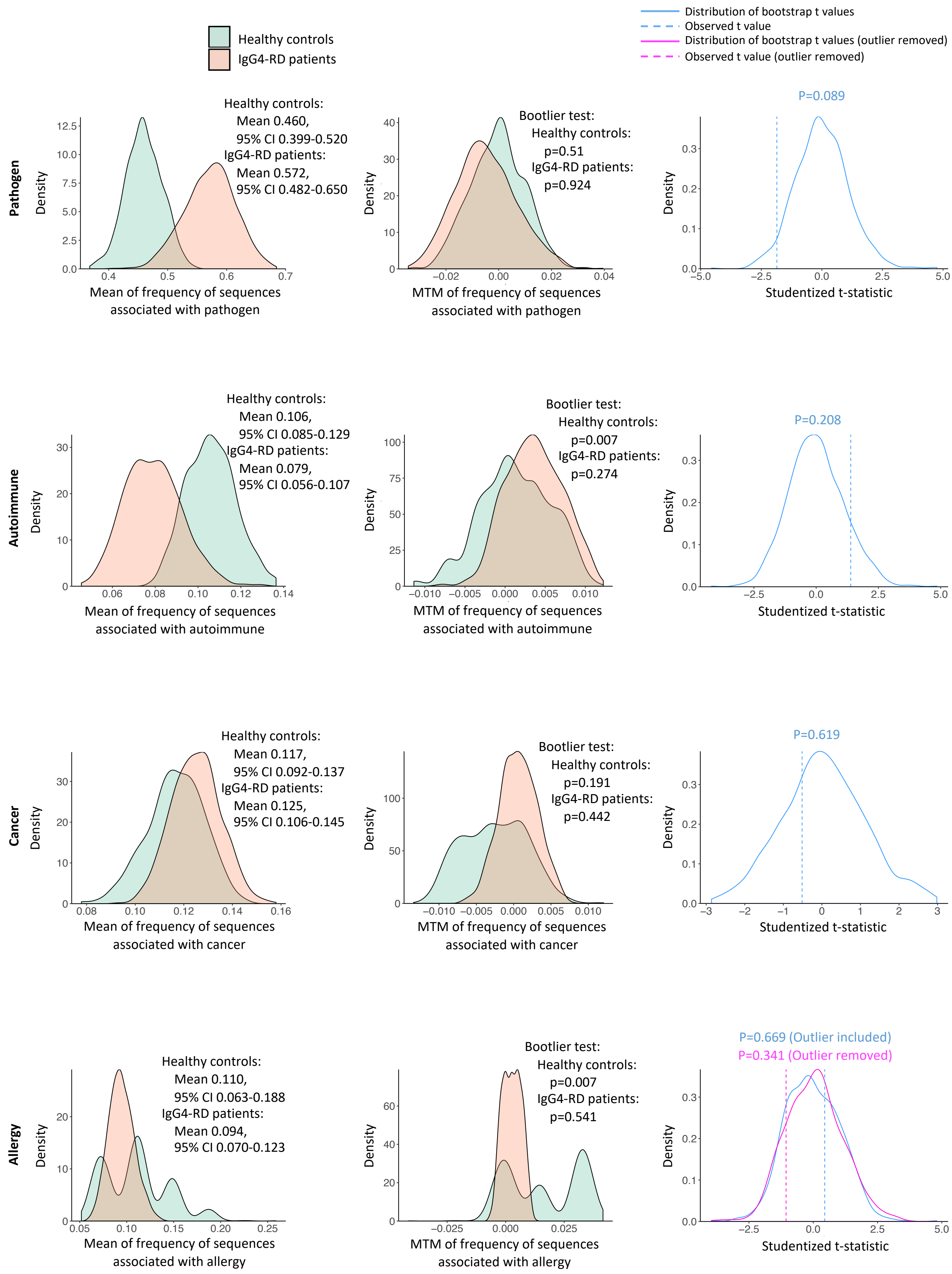

Healthy controls
IgG4-RD patients
Distribution of bootstrap t values
Observed t value
Distribution of bootstrap t values (outlier removed)
Observed t value (outlier removed)
Pathogen
Healthy controls: Mean 0.460, 95% CI 0.399-0.520
IgG4-RD patients: Mean 0.572, 95% CI 0.482-0.650
Mean of frequency of sequences associated with pathogen
Bootlier test: Healthy controls: p=0.51 IgG4-RD patients: p=0.924
MTM of frequency of sequences associated with pathogen
P=0.089
Studentized t-statistic
Autoimmune
Healthy controls: Mean 0.106, 95% CI 0.085-0.129
IgG4-RD patients: Mean 0.079, 95% CI 0.056-0.107
Mean of frequency of sequences associated with autoimmune
Bootlier test: Healthy controls: p=0.007 IgG4-RD patients: p=0.274
MTM of frequency of sequences associated with autoimmune
P=0.208
Cancer
Healthy controls: Mean 0.117, 95% CI 0.092-0.137
IgG4-RD patients: Mean 0.125, 95% CI 0.106-0.145
Mean of frequency of sequences associated with cancer
Bootlier test: Healthy controls: p=0.191 IgG4-RD patients: p=0.442
MTM of frequency of sequences associated with cancer
P=0.619
Allergy
Healthy controls: Mean 0.110, 95% CI 0.063-0.188
IgG4-RD patients: Mean 0.094, 95% CI 0.070-0.123
Mean of frequency of sequences associated with allergy
Bootlier test: Healthy controls: p=0.007 IgG4-RD patients: p=0.541
MTM of frequency of sequences associated with allergy
P=0.669 (Outlier included)
P=0.341 (Outlier removed)
Density
